# Supplementary material for: On-chip beam rotators, adiabatic mode converters, and waveplates through low-loss waveguides with variable cross-sections
Source: Light Sci Appl. 2022 Jul 7;11:214. doi: 10.1038/s41377-022-00907-4 (PMC9263149; doi:10.1038/s41377-022-00907-4)
Supplement: Supplementary file 1 — SUPPLEMENTAL MATERIAL [file 41377_2022_907_MOESM1_ESM.pdf]

Supplementary Information

On-chip beam rotators, adiabatic mode converters, and waveplates through low-loss waveguides with variable cross-sections

Bangshan Sun[1*], Fyodor Morozko[2], Patrick S. Salter[1], Simon Moser[3], Zhikai Pong[1], Raj B. Patel[4,5], Ian A. Walmsley[4], Mohan Wang[1], Adir Hazan[2], Nicolas Barré[3], Alexander Jesacher[3,6], Julian Fells[1], Chao He[1], Aviad Katiyi[2], Zhen-Nan Tian[7], Alina Karabchevsky[2*] and Martin J. Booth[1,6*]

[1]Department of Engineering Science, University of Oxford, Oxford OX1 3PJ, United Kingdom
[2]School of Electrical and Computer Engineering, Ben-Gurion University of the Negev, P.O.B. 653, Beer-Sheva, 8410501, Israel
[3]Institute of Biomedical Physics, Medical University of Innsbruck, Müllerstraße 44, 6020 Innsbruck, Austria
[4]Ultrafast Quantum Optics group, Department of Physics, Imperial College London, London, United Kingdom
[5]Department of Physics, University of Oxford, Oxford, United Kingdom
[6]Erlangen Graduate School in Advanced Optical Technologies (SAOT), Friedrich-Alexander-University Erlangen-Nürnberg, Paul-Gordan-Straße 6, 91052 Erlangen, Germany
[7]State Key Laboratory of Integrated Optoelectronics, College of Electronic Science and Engineering, Jilin University, Changchun 130012, China

*Corresponding to: Bangshan Sun (bangshan.sun@eng.ox.ac.uk), or Alina Karabchevsky (alinak@bgu.ac.il), or Martin J. Booth (martin.booth@eng.ox.ac.uk))

Note 1. Two different fabrication regimes of fs-laser written waveguides

Historically, fs-laser written waveguides have been created in two different fabrication regimes: a non-heating regime with low laser repetition rate (< 10 kHz); and a heating regime with high laser repetition rate (> 500 kHz). These two fabrication regimes involve different processes in creation of the RI profile for waveguides[1,2]. Here we develop technology based on fabrication in the heating regime. The major reason concerns the fabrication efficiency. Creating large size cross-section waveguides requires tens or even hundreds of laser scans. For fabrication in the non-heating regime, the single laser scanning speed has to be very low (0.02-0.1 mm/s) to make the RI modifications continuous. With heating regime fabrication, we are able to translate laser scanning with a speed of 8 mm/s, reducing fabrication time for one waveguide from several hours to 1-2 minutes. Another reason concerns refractive index modification, which is about $1\text{-}5\times10^{-3}$ with fabrication in the non-heating regime. This is not sufficient for confining modes in longer wavelengths such as telecommunication bands, while heating regime fabrication could provide us a much higher refractive index modification, leading to much better confinement of the mode profile for longer wavelengths.

Note 2. Polarization rotation and Spectral oscillation

We have mentioned in the main text that there are two individual phenomena which are explained by two different model. Firstly, we note that the root cause of polarization rotation is solely contributed by the adiabatic twisting of waveguide cross-section. In our case, the waveguide twisting itself is sufficient to produce polarization rotation.

Secondly, the spectral oscillation of polarization rotation is another phenomenon which is caused by the interaction of multiple modes. Here, we provide a mathematical representation of multimode inference, which contributes to the spectral oscillation.

Consider two modes characterised by propagation constants β_1 and β_2 and electric fields $E_1 = e_1(x,z)exp(i\beta_1 y)$, $E_2 = e_2(x,z)exp\,(i\beta_2 y)$. The total power of the two modes at the output facet is proportional to $|E_1 + E_2|^2 = |e_1|^2 + |e_2|^2 + 2Re\{e_1 \cdot e_2 exp\,(i\Delta\beta L)\,\}$, where $\Delta\beta = \beta_1 - \beta_2$ and L is the total length of the waveguide.

The last term $\Delta\beta L$ is 2π periodic and is responsible for the spectral oscillations. The observed oscillations of period $\Delta\lambda$ correspond to the phase change by 2π:

$$(\Delta\beta(\lambda + \Delta\lambda) - \Delta\beta(\lambda))L = \pm 2\pi.$$

Expanding $\Delta\beta(\lambda + \Delta\lambda)$ in Taylor series to the first order we get

$$\Delta\lambda \frac{d}{d\lambda}\big(\Delta\beta(\lambda)\big)L = \pm 2\pi.$$

With the definition of the modal group index[3] $n_g = -\frac{\lambda^2}{2\pi}\frac{d\beta}{d\lambda} = n_{eff} - \lambda\frac{dn_{eff}}{d\lambda}$, where n_{eff} is effective refractive index, we obtain the following expression for the period of spectral oscillations in either wavelength $\Delta\lambda$ or wavenumber Δk:

$$\Delta\lambda = \pm\frac{\lambda^2}{(n_{g1} - n_{g2})L}$$

$$\Delta k = \pm\frac{2\pi}{(n_{g1} - n_{g2})L}$$

Both n_g and n_{eff} depend only weakly on λ, so that $\Delta\lambda$ is approximately proportional to λ^2, and Δk is almost constant.

Figure S1. Choice of Zernike mode phases and their focal effects

We evaluated the possibility of using low order Zernike modes to control laser focal intensity distribution, in order to manipulate the refractive index profile of fabricated waveguides. Here we use Noll's sequential indices scheme for description of Zernike modes. Major Zernike modes, including primary astigmatism (Z5 and Z6), primary coma (Z7 and Z8), primary trefoil (Z9 and Z10), primary spherical (Z11) were considered. Simulations of focal intensity distributions with these phases introduced are presented in Fig. S1. To better control laser processed refractive index modification, the focal intensity should be circularly symmetric in the x-y plane, normal to the optical axis. As shown in Fig. S1, Zernike modes from Z5 to Z10 all affect the focal symmetry in the x-y plane. The Z11 phase is the only one that preserves symmetry as compared to an un-aberrated focus. In addition, by introducing a negative Z11 phase, focal energy shifts to the bottom part of the laser focus which is in favour of heat accumulation in refractive index modification of glass. We experimentally found that this resolves the complicated negative / positive refractive index regions processed by a laser focus in conventional direct laser fabrication. We note that higher order spherical aberration phases such as Z22 and Z37 may also be suitable for controlling the fs-laser refractive index modification in glass, as might other rotationally symmetric phase patterns.

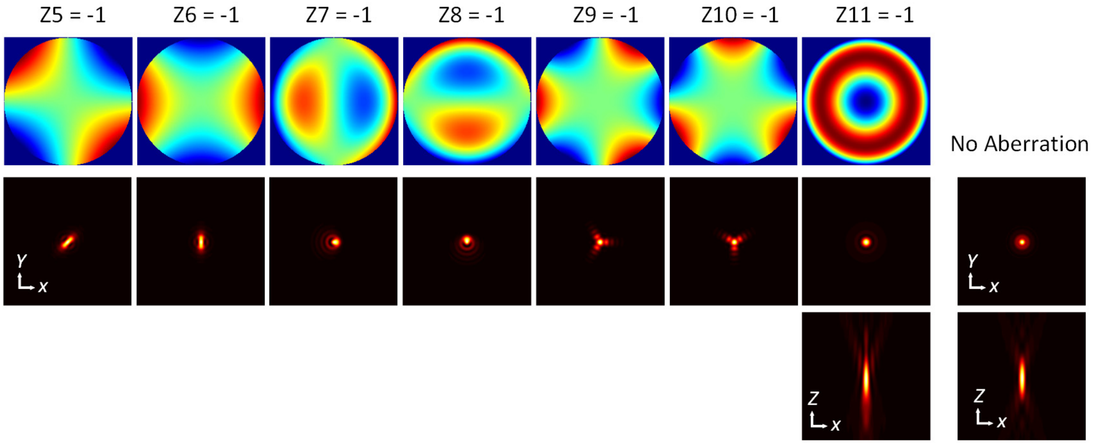

Figure S1. Top: 2D plot of important Zernike polynomials (Z5 to Z11) with amplitude of -1 radian rms. Sizes of intensity profile are 15×15µm (size is half of the images in main text). Middle: Simulated focal intensity distribution of laser focus in the x-y plane (2µm below nominal focal point), with either Zernike phase Z5 to Z11 introduced, or no phase aberration. Bottom: Simulated focal intensity distribution of laser focus in the x-z plane, where z is the optical axis, with either Zernike mode Z11 introduced, or no phase aberration. The simulation assumes 0.5NA objective lens with 514 nm input laser.

Figure S2. Classic multiscan vs SPIM-WGs fabrications with varying core spacing

We investigated fabrication of waveguides with smaller and larger core spacing. Both *x*-aligned and *z*-aligned configurations were investigated, and both LED illuminated microscopic images and 785nm laser mode profiles were recorded. As Fig. S2 shows, the SPIM-WGs technique created much better waveguides than classic multiscan technique for all the core spacings.

We would like to particularly point out that classic multiscan in the heating regime is not able to create any functional waveguides with designed core spacing <2 µm. In such cases, the structures created by classic multiscan cannot be categorized as “waveguides” due to uncontrollable and unexpected cross-sections and poor laser guiding. SPIM-WGs enabled this new capability by providing uniform cross-sections and waveguide modes with designed core spacing <2µm.

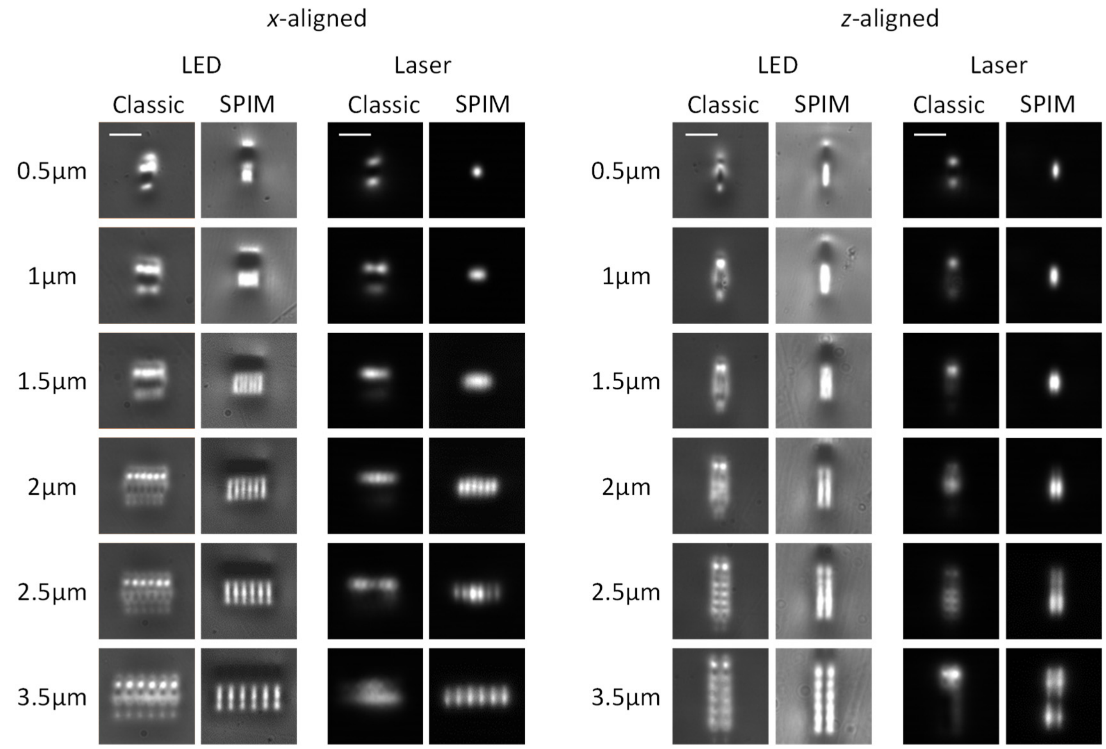

Figure S2. Comparison of heating regime classic multiscan fabrication with SPIM-WGs fabrication for waveguides with varying core spacing (from 0.5µm to 3.5µm). The waveguides are 6×2 cores. The left two columns are *x*-aligned and the right two columns are *z*-aligned. Both LED illuminated microscopic images and 785nm laser mode profiles are recorded and compared. The waveguide with larger than 2µm core spacing has multimode transmission. Scale bars are 10 µm.

Figure S3. Comparison of waveguides input facets and output modes

The input facets and output modes of single-scan waveguides and SPIM-WGs are compared in Fig. S3. Though the refractive index modification is different between these two, single scan waveguides with Z11=0 and Z11=-1 have comparable mode profiles. LED images show the fabricated structure matches well to our design. Larger cross-sections of 20×4μm and 10×4μm support multiple modes when guiding 785nm laser. It is clear that twisted waveguides provide excellent capability in beam rotation for both 20×4μm and 10×4μm cross-section sizes.

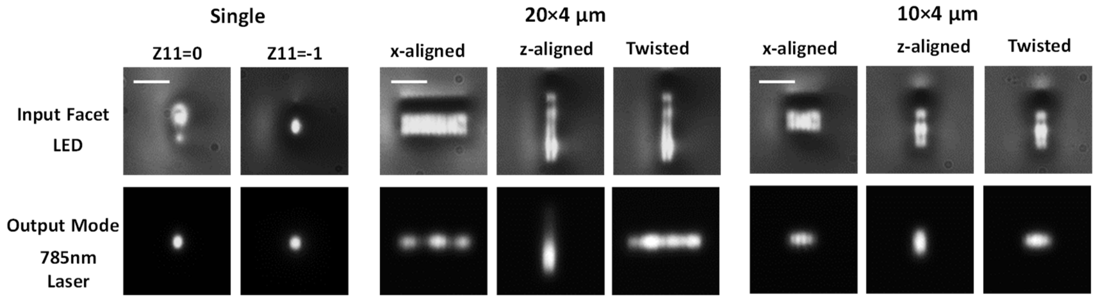

Figure S3. LED illuminated microscopic images of input facet (top row), and 785nm laser guiding mode profiles of waveguide output (bottom row) for waveguides with different cross-section shapes and sizes. From left to right: classic single-scan with either Z11=0 or Z11=-1; *x-aligned*, *z-aligned*, twisted waveguides with cross-section size of 20×4μm; *x-aligned*, *z-aligned*, twisted waveguides with cross-section size of 10×4μm. In the case of multi-modes, the major mode is presented. Twisted waveguides hence twisted section length of 1.4mm. Scale bars are 10 μm.

Figure S4. Refractive index profiles for single scan, multi-scan SPIM-WGs

As shown in Fig. S4, the refractive index profile of single scan SPIM-WG with Z11 = -1 reveals comparable shape with the simulated laser focal intensity profile in Fig. S1. In comparison, for a classic single-scan waveguide, the waveguide cross-section usually has a fairly different shape compared to laser focal spot (Fig. 1 (d)). Adding additional spherical aberration phase to the fabrication simplified refractive index profile. When the number of scans increases, the positive refractive index becomes even higher, and negative refractive index region gradually builds up in the surrounding area. In addition, we can see that the positive refractive index from the multi-scan smoothly overlaps with each other along vertical direction.

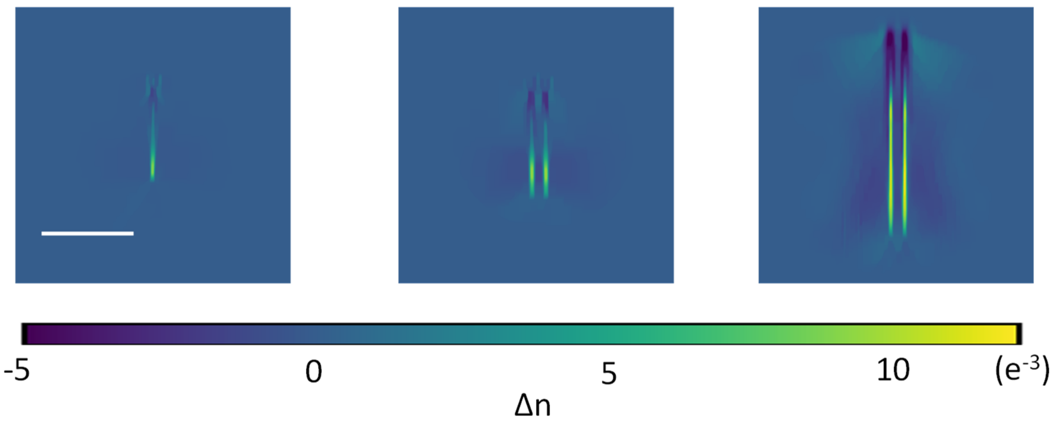

Figure S4. Measured refractive index profiles. Left: SPIM-WG with a single core. Middle: SPIM-WG with two cores, with 1.5 μm core spacing distance. Right: SPIM-WG with 9×2 cores, with 1.5μm core spacing distance. Scale bar is 10 μm.

Figure S5. Waveguide characterization systems

Fig. S5 shows diagrams of two waveguide characterization systems. Details are included in main text "Materials and methods": "Waveguide images and loss characterizations" and "Waveguide polarization measurements".

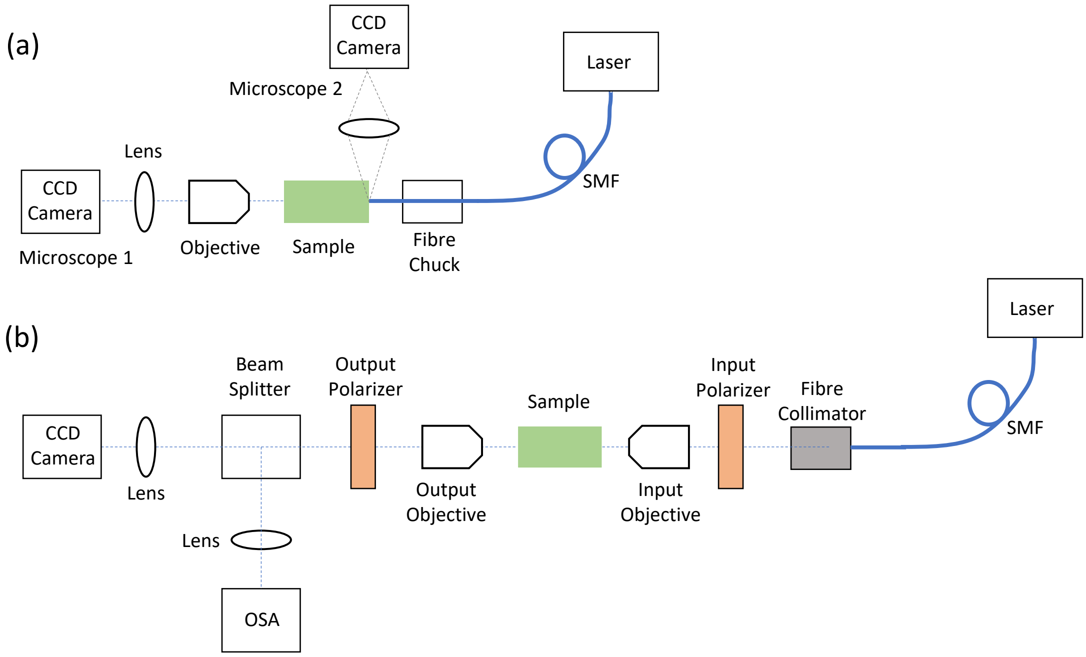

Figure S5. (a) The system to image waveguide facets, obtain laser mode profiles, and measure waveguide losses. There were two home-built microscopes: microscope 1 is used to obtain waveguide facet images and mode profiles; microscope 2 was used for live monitoring and measurement of the distance between input fibre tip and waveguide facet. (b) The system to characterize the polarization rotation effect for the waveguides.

Figure S6. Coupling losses of various cross-sections from a large size input beam

We measured the coupling losses from an expanded input beam to the waveguides described in Fig. 3 (a). The input beam was expanded to be ~13.2µm in diameter by keeping a 34 µm distance of the fibre tip and waveguide facet. We evaluated the coupling losses for all types of waveguides, with results summarised in Fig. S6. All waveguides have large coupling losses as the waveguide facets only covered a small portion of the input beam. We found the three sets of waveguides with input cross-section of 20×4 µm have slightly lower coupling losses. This is due to a much bigger waveguide facet, bringing more overlap with input beam, therefore reducing coupling losses even in the presence of significant mode mismatching. The reason that *x-aligned* rectangular waveguides have lower coupling losses than that of *z-aligned* ones is due to the *x-aligned* facet having slightly larger area (Fig. 2 (b) in main text). The *z-aligned* 10×4 µm waveguide had slightly higher coupling loss than single-scan waveguides, where we think is due to single-to-multiple mode mismatch. These high coupling losses were reduced to be in the range of 1 to 2 dB when bringing the fibre tip infinitely close to the sample facet. In the main text, we experimentally demonstrated that the coupling loss of SPIM-WGs can be significantly reduced to around 0.2dB by advanced mode matching.

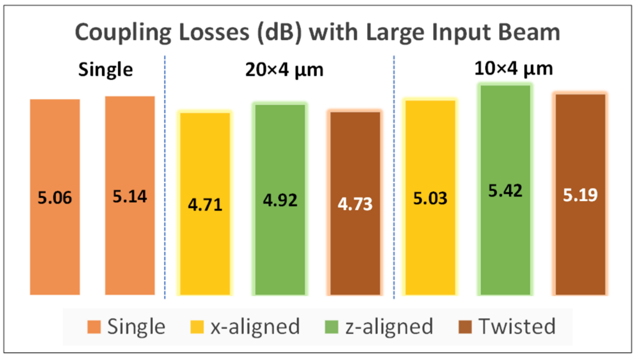

Figure S6. Coupling losses of the waveguides from an expanded Gaussian input beam with diameter of ~13.2µm at 785 nm wavelength. The detailed waveguide cross-sections are described in Fig. 3 (a) of main text.

Figure S7. Demonstration of adiabatic process for mode conversion

We experimentally investigated the adiabatic and non-adiabatic regime of the mode converters by measuring the insertion loss (also called total loss = propagation loss + coupling loss) for mode converters with varying conversion lengths. The conversion length is defined as the length of the waveguide where the cross-section is changing from one shape/size to anther shape/size. As the below figure shows, the insertion losses stay low and do not have considerable change for longer length of conversion region. However, when the conversion region was shorter, the loss dramatically increased. There is a cut-off length between high loss and low loss, which also distinguishes the boundary between non-adiabatic and adiabatic regimes. Based on the experimental observation, the cut-off length is around 20 µm, 25 µm, 250 µm for circular-rectangular, circular-ppKTP and circular TE00-TE01 converters, respectively.

We also observed that the insertion loss decreased when the conversion length approaches zero. We believe this is due to fact that extra loss = loss from high order mode excitation + loss from radiation modes. In the non-adiabatic regime, the loss from the radiation modes happens all the way along the waveguide conversion region, so that the radiation modes loss may decrease with smaller conversion length. When the conversion length approaches zero, the loss from radiation modes may be lower which contributes to the reduction of the total loss. However, Fig. S7 already demonstrated that the mode converters we reported in the main text with conversion length of 1.4mm operate in the adiabatic regime, as we only see the loss increment for much shorter conversion lengths.

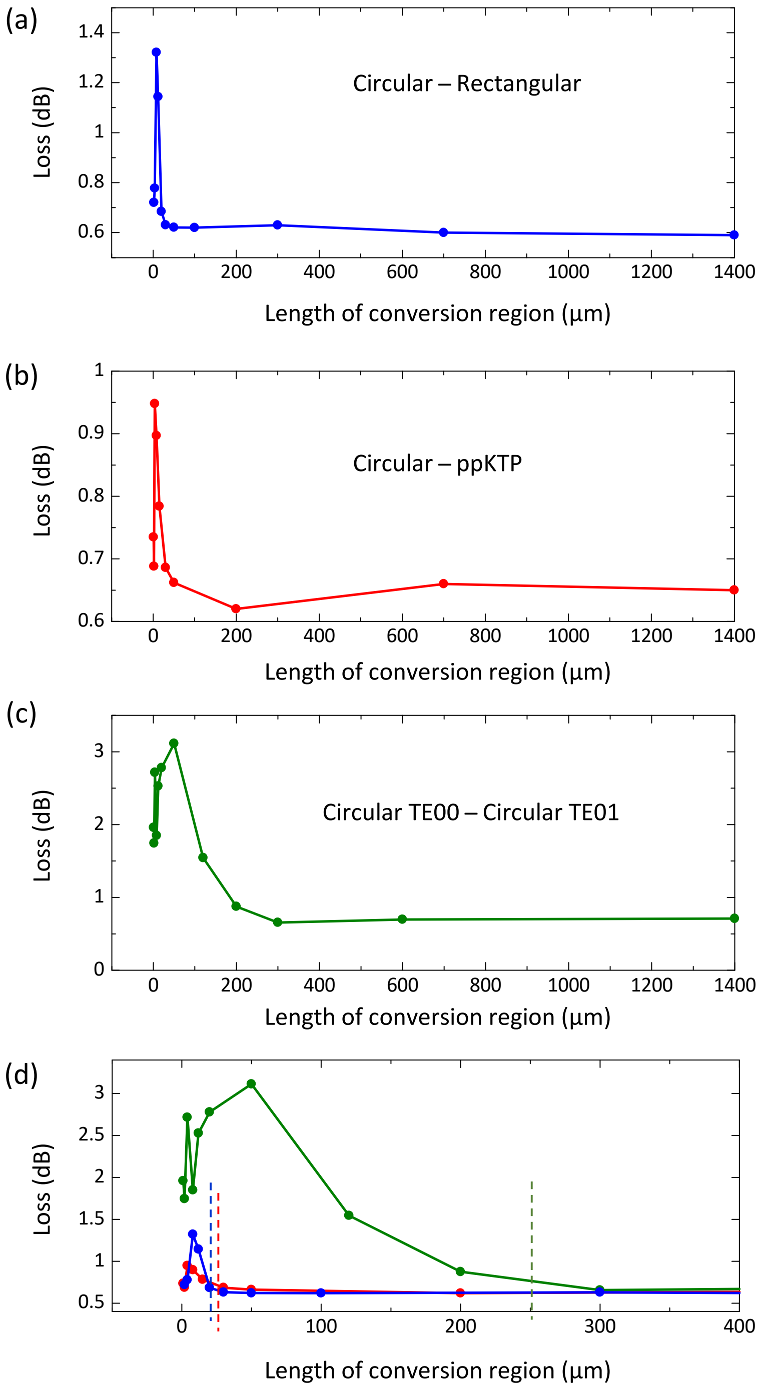

Figure S7. Demonstration of adiabatic process for mode conversion. The plots are total insertion losses of the mode converters versus length of conversion region. (a)(b)(c) study of three different types of mode converters. (d) Comparing three curves in one figure for the shorter length of conversion region. Our mode converters in main text were fabricated with 1.4mm conversion length which is obviously in the adiabatic regime.

Figure S8. Polarization conversion of 90-degree twisted SPIM-WGs versus wavenumber of the transmitted laser light

Here is a plot of polarization conversion versus wavenumber for the waveguides presented in Figure 5 of the main text. The oscillation periods almost stay a constant period in terms of Δk (Supplementary Note 2).

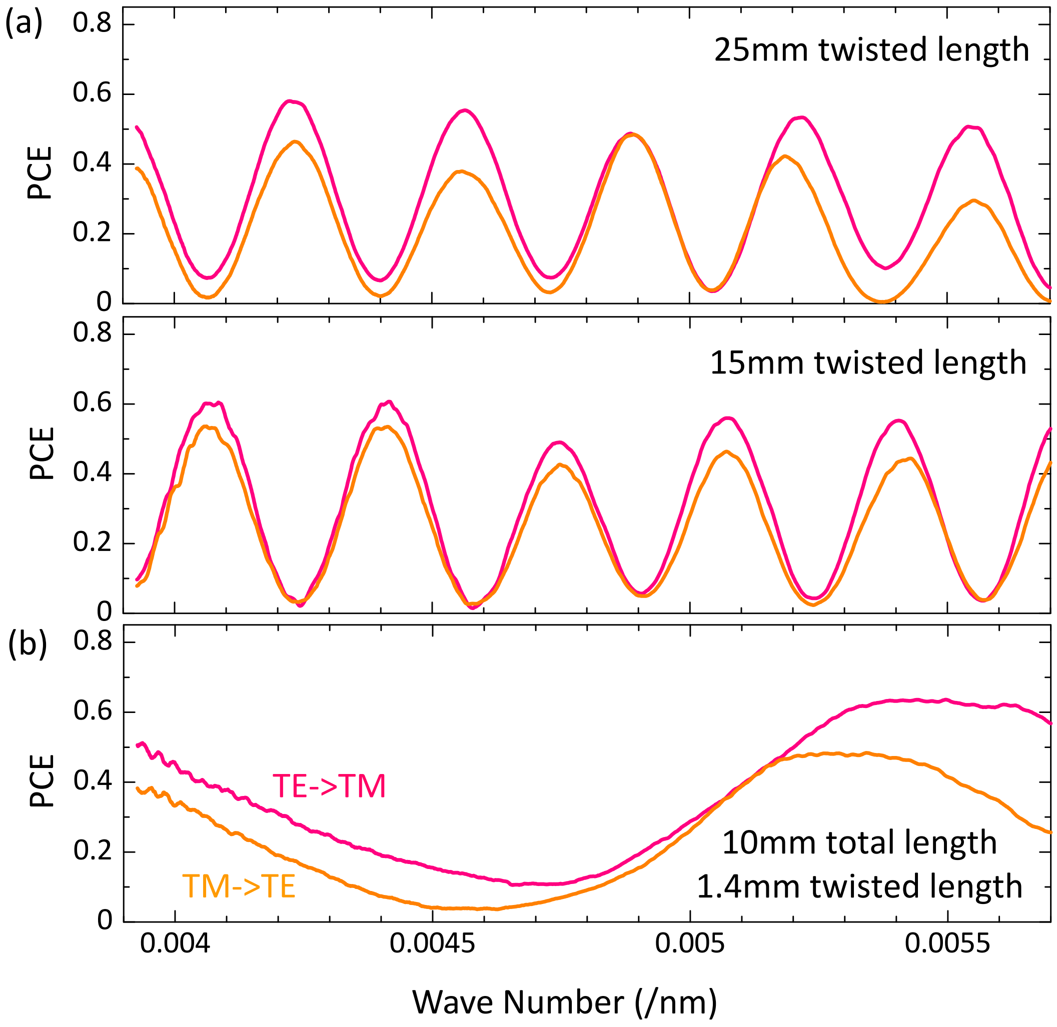

Figure S8. Measured broad-band polarisation conversion versus wavenumber of tested laser light for 90-degree twisted waveguides. (a) Total length of waveguides is 30mm. (b) Total waveguide length is 10mm.

Figure S9. Broadband polarization conversion for straight rectangular SPIM-WGs without twisting

We measured the polarization conversion for straight rectangular waveguides, in order to compare with twisted waveguides (Figure 5 in the main text). As demonstrated by Figure S9, the straight rectangular waveguides have nearly zero polarization conversion across most wavelengths. Regarding the birefringence, mode calculations for the rectangular waveguides show that the waveguide birefringence $\delta = n^{TE}_{eff} - n^{TM}_{eff}$ is equal to approximately 10^{-5} for TE and TM modes of the same order. This corresponds to the beat length[3] $L_{beat} = \lambda/\delta$ of 154 mm at the wavelength λ=1550 nm. The lengths of rectangular section in our waveguides are far below the beat length, thus the birefringence can be safely neglected in our configuration.

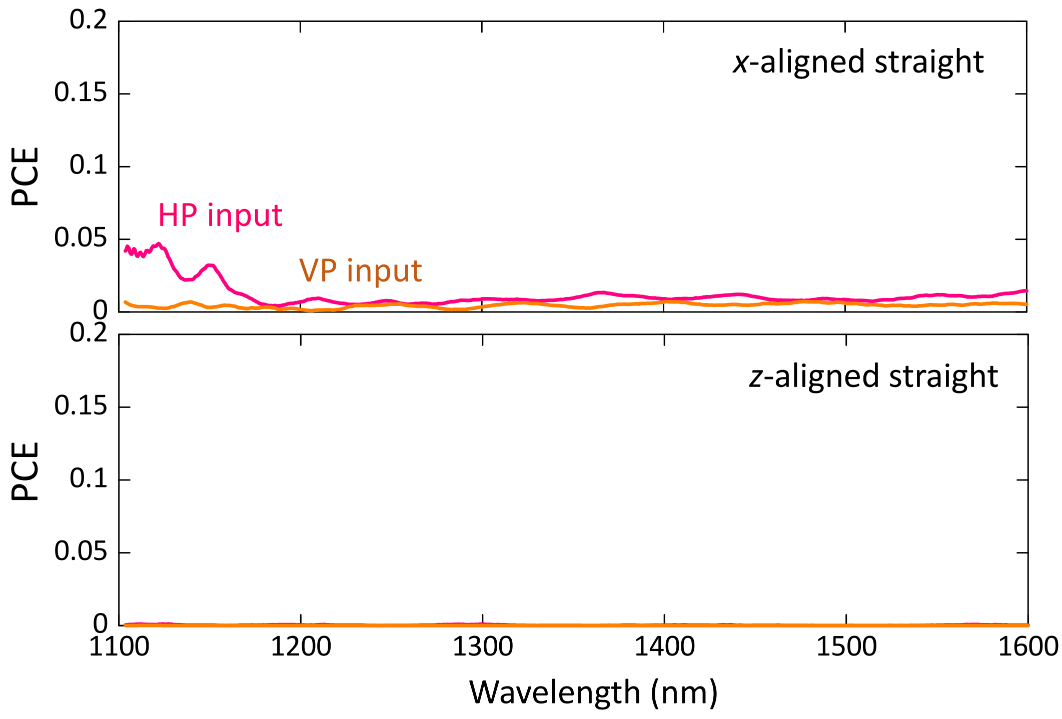

Figure S9. Measured broad-band polarisation conversion versus wavelength of tested laser light for straight rectangular waveguides with total length of 30mm. Note the vertical axis has different range with Figure 5 of main text. Top: *x-aligned* straight rectangular waveguide. Bottom: *z-aligned* straight rectangular waveguide.

Figure S10. Microscopic images of SPIM-WG facets with larger field of view

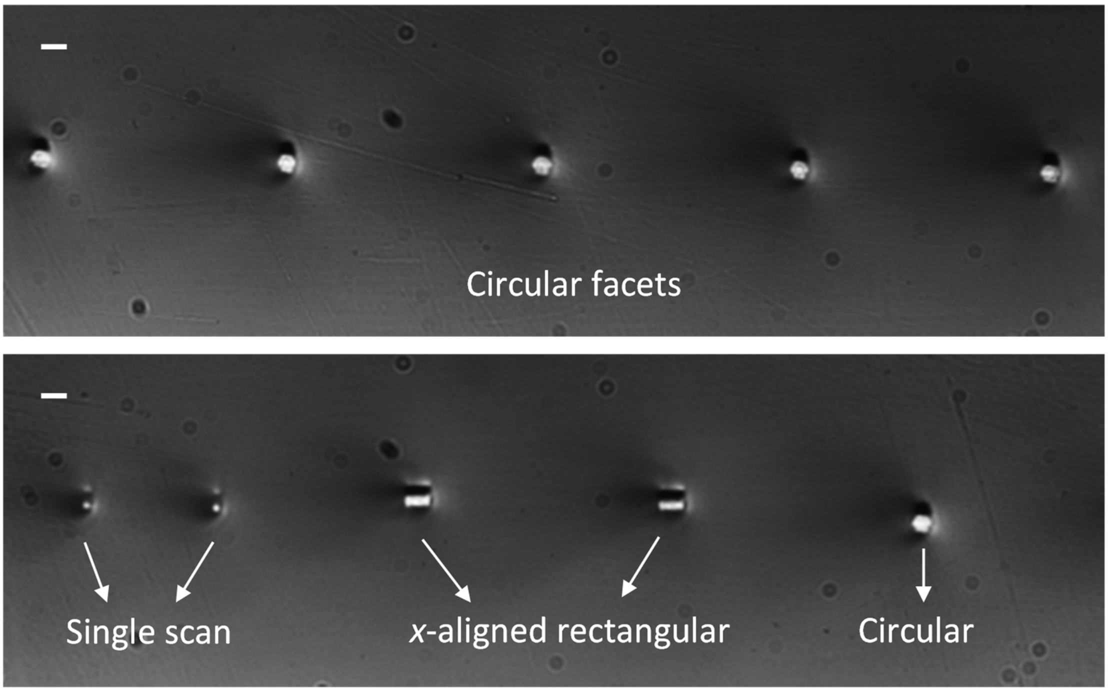

Figure S10. Larger field of view microscopic images of various SPIM-WG facets. Top: circular waveguide facet matching to an 8µm single mode fibre. Bottom: comparison of single scan waveguide with Z11=-1, *x-aligned* rectangular facet of twisted SPIM-WGs, circular facet of a mode converter. Scale bars are 10 µm.

Figure S11. Diagram highlights new capabilities enabled by SPIM-WGs

We would like to highlight that SPIM-WGs enabled two new capabilities that thermal regime classic multiscan is not capable to do. These are: 1) to create cross-section variable waveguides embedded in glass with high refractive index (RI) contrast (> 0.015); 2) to create precisely controllable small size (0.5 - 5µm) cross-section variable waveguides for high-quality mode converters. The comparison of capabilities is summarized in Fig. 6.

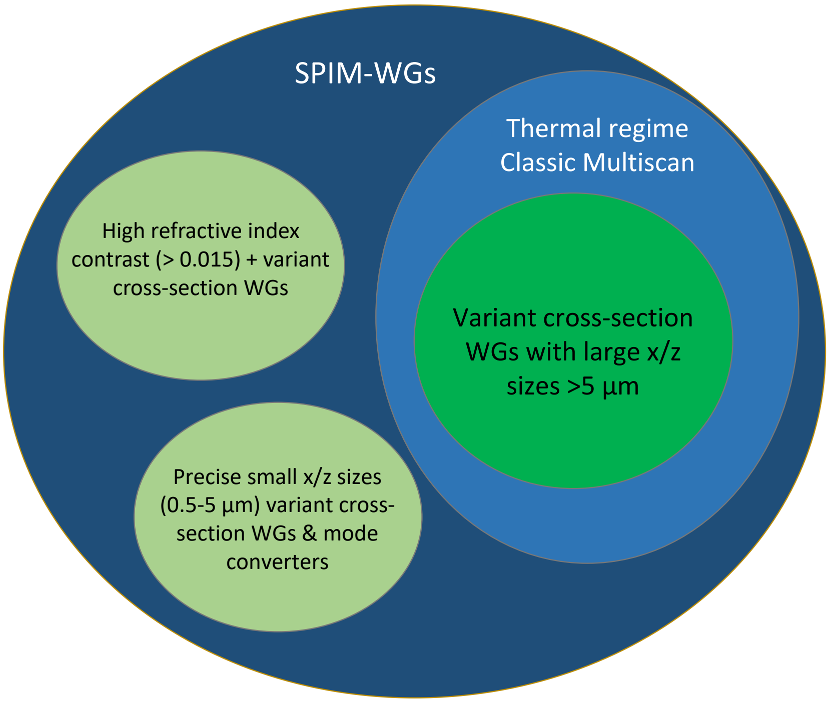

Figure S11. Diagram highlights new capabilities enabled by SPIM-WGs. There are two new capabilities demonstrated in this paper that thermal regime classic multiscan is not capable to do. Note the non-heating regime classic multiscan is not included here due to dramatically low efficiency (orders of magnitude slower).

References

1. Valle, G. Della *et al.* Micromachining of photonic devices by femtosecond laser pulses. *J. Opt. A Pure Appl. Opt.* **11**, 13001 (2009).

2. Gross, S. & Withford, M. J. Ultrafast-laser-inscribed 3D integrated photonics: Challenges and emerging applications. *Nanophotonics* **4**, 332–352 (2015).

3. Snyder, A. W. & Love, J. D. *Optical waveguide theory*. (Chapman and Hall, 1983).
